# Measuring Adherence in Hypertensive Patients—Pilot Study with Self-Efficacy for Appropriate Medication Use Scale in Bulgaria

**DOI:** 10.3390/medicina61030478

**Published:** 2025-03-09

**Authors:** Zornitsa Mitkova, Elena Dimitrova, Velislava Kazakova, Nikolay Gerasimov, Diyan Gospodinov, Javor Mitkov, Stamen Pishev, Guenka Petrova

**Affiliations:** 1 Department of Organization and Economy of Pharmacy, Faculty of Pharmacy, Medical University Sofia, 1000 Sofia, Bulgaria; avakazakova01@gmail.com (V.K.); guenka.petrova@gmail.com (G.P.); 2 National Cardiology Hospital Sofia, 1309 Sofia, Bulgaria; elena.sv@gmail.com; 3 Medical College, Trakia University, 6000 Stara Zagora, Bulgaria; nikolay.gerasimov@trakia-uni.bg; 4 Medical Center “Staykov” Burgas, 8000 Burgas, Bulgaria; diyan_gospodinov@abv.bg; 5 Department of Pharmaceutical Chemistry, Faculty of Pharmacy, Medical University Sofia, 1000 Sofia, Bulgaria; y.mitkov@pharmfac.mu-sofia.bg; 6 Diagnostic-Consulting Center I-Burgas, 8000 Burgas, Bulgaria; s_pishev@abv.bg; 7 Scientific Research Institute “InnoMedSci”, Medical University Sofia, 1431 Sofia, Bulgaria

**Keywords:** adherence, hypertension, Bulgarian patients, SEAMS

## Abstract

*Background and Objectives*: Hypertension is found as the leading cardiovascular disease in Bulgaria and the most frequent lifelong condition with a high risk of non-adherence. The aim of this study is to assess the hypertension patients’ adherence to medication therapy in Bulgaria by using the self-efficacy for appropriate medication use scale (SEAMS). *Materials and Methods*: A cross-sectional study was conducted in the major cardiology settings in the capital Sofia and in Burgas, the fourth largest city in Bulgaria. Data were collected from January 2024 to July 2024. The SEAMS was validated in Bulgarian in a sample of patients with hypertension that made it extremely suitable for our study. We applied 19 questions. Additionally, we used Cronbach’s α, ANOVA analysis, Pearson correlation, and covariance tests for statistical significance. *Results*: A total of 232 patients completed the study. Both genders are almost equally distributed (49.6% male and 50.4% female). The results reveal that the increase in the number of diseases and prescribed medicinal products leads to a worsening adherence level. Advancing age negatively affects the adherence scores. The study showed that in half of the age groups studied (patients’ age: 40–49, 60–69, and 70–79), adherence in patients treated with FDCs was better or almost the same (patients’ age: 50–59) as those treated with several mono-products. *Conclusions*: Measuring adherence with SEAMS in the hypertensive Bulgarian population in two regions reveals a good level of adherence. Factors negatively affecting adherence were older age, polypharmacy, co-morbidity, and therapy with monoproducts.

## 1. Introduction

Adherence is perceived as “the degree to which a person’s behaviour regarding medications, diet, or required lifestyle changes coincides with the medical instructions” [1]. Adherence to medication therapy describes the corresponding individual’s behaviour to take medicines as prescribed [2].

Factors influencing adherence are related to the socio-economic environment, conditions, patients, healthcare system, and therapy [3]. Hypertension is the leading cardiovascular disease in Bulgaria and the most frequent lifelong condition with a high risk of non-adherence [4]. The World Health Organization (WHO) estimated that 71% of women and 57% of men aged 30–79 in Bulgaria suffer from hypertension, but only 23% of them control the disease [5], a fact that presumes a high level of non-adherence. In order to reach controlled disease in 50% of hypertension patients, more than 621,000 people with hypertension need to be effectively treated. If the disease is adequately controlled, 76,000 deaths could be prevented by 2040.

A lower level of adherence is found in patients from low-income countries taking a large number of medicines per day, non-informed or with little knowledge of hypertension-related complications, and with the available depressive symptoms. The main potential risk factor for uncontrolled hypertension and complications is the lack of adherence to prescribed medicines [6]. Often, non-adherence to therapy is followed by uncontrolled blood pressure and an increasing rate of complications, hospitalization, and mortality [7].

Nearly 10 million deaths were directly attributable to non-adherence to antihypertensive therapy in 2019 [8]. Measuring adherence is a challenge for researchers. The self-efficacy for appropriate medication use scale (SEAM) was developed to measure perceived non-adherence in patients with chronic diseases, first tested for hypertension, and validated in many countries [9].

The aim of this study is to assess the hypertension patients’ adherence to medication therapy in Bulgaria by using SEAMS.

## 2. Materials and Methods

A cross-sectional study was conducted in cardiology outpatient settings in the capital Sofia and in Burgas, the fourth largest city in Bulgaria. Data were collected from January 2024 to July 2024.

Patients were interviewed during their regular examination by cardiologists in the offices. Physicians instructed patients on how to respond to each set of items, oriented them for the response choices, and allowed the patient to indicate a response verbally or by pointing to their desired choice. We applied the SEAMS, developed and validated by Risser et al. The SEAMS was developed by a multidisciplinary team via patient interviews to provide a valuable assessment of medication self-efficacy in patients with coronary heart disease and other co-morbid conditions, validated for measurement of self-efficacy and suitability for several levels of patient literacy [10].

### 2.1. Inclusion and Exclusion Criteria

Participants were Bulgarian adults who signed a consent form. Patients over 18 years old and mentally capable of responding with clinically proven hypertension as a primary diagnosis were recruited in the selected object at random. Every hypertensive patient visiting the office during the examination day who agreed to participate was interviewed until we collected the necessary sample. After obtaining consent from the patients, they were invited to complete the questionnaire with the physicians’ assistance.

### 2.2. Sample Size

We used the Raosoft calculator for sample size calculation [11]. The number of patients required for a representative sample is 270; however, a total 232 participants were recruited. Therefore, we consider the survey as a pilot for the population in both cities. The rate of response is 100% due to active cardiologists’ engagement in the patient’s recruitment and examination.

### 2.3. Data Collection

During the study, patients filled out the SEAMS questionnaire as described above with support from their cardiologist. Additionally, we recorded the patients’ age, education level, number of diseases, and number of prescribed medicines, including mono- or fixed-dose combination therapy.

### 2.4. Data Analysis

The SEAMS was validated in the Bulgarian language for a sample of patients with hypertension, which made it extremely suitable for our study. We applied 19 questions with three possible answers each: yes, completely (3 points); sometimes (2 points); or no, not at all (1 point)—Appendix A. If a patient gave the answer “no, not at all” to all questions, he/she received a score of 19 (corresponding to worst possible adherence) and if they answered “yes, completely” to all questions, they received a maximum score of 57 (corresponding to the highest possible adherence). A score of 38 is assumed as borderline between sufficient (above 38) and insufficient (below 38) adherence.

Reliability and Validity Testing

The final 19-item scale was tested for internal consistency reliability via Cronbach’s α coefficient. SPSS v. 19 was used for the calculations. The Cronbach’s α levels higher than 0.90 reveal an excellent reliability level [12].

The ANOVA single factor test was used to compare differences in adherence between men and women. The null hypothesis stated that there is a statistically significant difference between the explored variables (*p* < 0.05). We also consider the difference between F values, so if F < F crit, we accept the null hypothesis.

Pearson correlation was used for testing the correlation of age with adherence. Standard deviation (SD) was calculated per question depending on the answer of patients who participated. SD was also presented for each patient group, considering their gender, education, and co-morbidity. The covariance test was used for measuring the degree of variation between two parameters—number of prescribed medicines and final adherence score per patient. A negative covariance index indicates that as one variable increases, the other tends to decrease. If the covariance index is positive, such a relation does not exist.

### 2.5. Ethical Considerations

Ethical permission for the study was obtained from the Ethical committee of the Medical University Sofia (decision No. 51/25.01.2024).

## 3. Results

### 3.1. General Sample and Adherence Characteristics

A total of 232 patients completed the study. Both genders are almost equally distributed (49.6% male and 50.4% female). An average SEAMS score for both genders is also insignificantly different. The male score is 49.14 (SD 5.86), and the female score is 48.94 (SD 6.66) (Table 1). We may assume that gender does not affect adherence to medication therapy in our cohort. The ANOVA single-factor test compares the adherence level between both gender groups (*p* = 0.8444) and confirms our assumption for lack of significant differences in adherence to medication between male and female.

The adherence rate definitely decreases when the number of established diagnoses increases (Table 1). The number of medications prescribed can also be discussed as a factor influencing adherence to treatment. It was found to be highest in patients treated with one and three medicines (and also eight, which is an exception), but overall no clear association was found between the decrease in adherence and the increased number of medicines in our sample of patients. Therefore, other factors also influence medication adherence in hypertensive patients.

### 3.2. Factors Influencing Adherence

Participants with a secondary level of education prevail in the cohort (55.6%), but the adherence level is quite close between the groups. Even participants with higher education achieved lowest score of adherences (48.26), but the difference were not found statistically significant.

Overall, with the increase of the number of diseases and prescribed medicinal products, adherence worsens from 48.78 to 46.92 and from 50.84 to 51.5, respectively. Participants taking 9 or 11 medicines and having six and seven diseases were excluded from the analysis due to their small number in the sample (Table 1).

These results were confirmed from the covariance analysis (Table 2).Negative covariance coefficients are calculated for most of the questions, meaning that in such a situation, the increase in the number of prescribed medicines negatively influence adherence. Positive covariance indexes were calculated only in four cases—during the very busy day, in case of a change of therapy, accepting taking medicines as routine, and always remembering to take medicines. We can assume that in these four cases, despite the number of prescribed medicines, patients always feel not confident, so the adherence might decrease.

Internal consistency and reliability were evaluated using Cronbach’s alpha (0.928). The Cronbach’s alpha result indicates very good reliability and internal consistency of response values for each participant across a set of questions.

Correlation between age and adherence to medication therapy is −0.1616 (Pearson), pointing out the negative but weak correlation between age and adherence. This means that with the increase of age, adherence decreases. Further age subgroup analysis was performed by dividing the participants into nine age groups (Table 3). It is evident from Table 3 that with the increase in age, the number of diseases and medicines per individual increase, but the adherence to therapy decreases significantly. The group above 90 years of age is too small to comment on the meaning of the results.

The highest number of patients treated with fixed-dose combinations falls in the age groups 50–59 and 60–69. With advancing age, different fluctuations are observed in terms of adherence levels (Table 3). People above 50 years of age usually started to develop more than one chronic disease, and for them, combination therapy is unavoidable. Treatment with FDCs can make daily life easier, and it is the prerequisite for better adherence to treatment.

**Figure 1:**
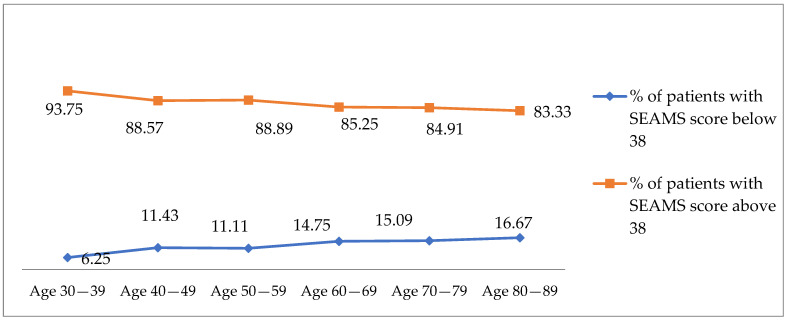
Percentage of patients with SEAMS score below 38 and above 38 per age group.

Around 83–93% of participants reported SEAMS scores above 38 (Figure 1), which confirms a relatively high level of adherence. The figure also points out that with the increase in the age, the SEAMS score decreases, meaning that adherence deteriorates.

## 4. Discussion

Our study is the first one applying SEAMS methodology to explore adherence in Bulgarian patients with hypertension. Despite a large number of scales examining the patients’ adherence, we selected SEAMS as it was created and validated for patients with cardiovascular diseases. The other advantage of SEAMS is that it was created for self-assessment of individuals with a broad range of literacy [13] and could evaluate lots of (n=19) situations in which adherence could be influenced by independent factors.

We examined 232 patients with different levels of education, age, gender, co-morbidities, and number of medications. Our results reveal that patient adherence decreases with an increasing number of diseases and number of prescribed medicines. The advancing age could also be discussed as a factor that negatively affects adherence scores. The exception is the 50–59 age group, which is among the patients with the highest level of FDCs therapy (63% of all patients in this age group) and the highest SEAMS score. The study showed that in half of the age groups studied (40–49, 60–69, 70–79 patients’ age), adherence in patients treated with FDCs was better or almost the same as those treated with several mono-products. In a previous study in Bulgaria, cardiovascular patients treated with FDCs reported higher adherence to treatment than those on monotherapy [14].

An online survey of Bulgarian patients shows that 61% take two or more than two tablets daily for hypertension therapy. A total of 63% of patients forget to take their medication, although 25% of respondents were informed of the risk of non-adherence [15]. Similar results reported that patients with co-morbid conditions are taking multiple medications, which is negatively associated with adherence to treatment (*p* < 0.009). Almost half of participants (49%) who reported lack of co-morbidities are adherent to antihypertensive therapy, whereas participants with one or more co-morbidities reported (41, 39%) lower adherence levels. Better knowledge of the disease, as well as for the complications, resulted in better adherence level and patients became seven times more likely to adhere to prescribed medicines [16].

In hypertension centers in the United Kingdom, it was found that 55% of patients are non-adherent to prescribed medicines and 20% of them are completely non-adherent. Non-adherence correlated with younger age, female sex, number of prescribed antihypertensive medicines, and total number of prescribed medicinal products (total pill burden) [17]. Our study results also found that pill burden negatively affected adherence. The SEAMS methodology applied in elderly patients with hypertension showed a moderate level of adherence and concluded that the age when the treatment was initiated is significantly associated with the level of medication adherence [18].Patients who reported occasional non-compliance with prescribing regimens are younger and experienced more adverse effects compared with those who were fully compliant [19].

The introduction of different educational programs and increasing the patients’ literacy could increase adherence to therapy [20,21]. Our study reveals younger patients in Bulgaria are more adherent. This result confirms the need for the development of educational strategies promoting adherence with an emphasis on older patients’ groups and long-term results of the therapy.

The average SEAMS adherence score for the Bulgarian population with hypertension in the observed group is 49 points from a maximum of 57, which we can consider as a good level of adherence. The proportion of patients with low adherence is about 12.9%, which is lower than results from previous studies (25%) [15]. On the other side, we found a high validity and consistency in the answers. Therefore, differences can only be explained with the tools for measuring adherence. In our opinion, SEAMS is built on more questions and evaluates a variety of practical situations that can influence adherence.

The study has some limitations. The first one is the sample size of participants. We could not ensure the necessary number of participants, and therefore we cannot consider our results generalized. The second limitation is that we observed patients from two regions; nevertheless, they cover almost 25% of the population, so our results are more relevant to those two regions. As a pilot study on the usability of SEAMS, we did not explore detailed patients’ characteristics, such as type of antihypertensive medicines, pregnancies, or other medical or demographic categories. Additional studies are needed to deeply explore the SEAMS feasibility for adherence measurement and associated factors.

## 5. Conclusions

The use of SEAMS to measure adherence in hypertensive patients in Bulgaria in two regions shows a good level of adherence. Factors that negatively influence adherence are higher patient age, polypharmacy, and co-morbidities. The present study found no difference in adherence between the two genders and patient education. Further studies, including participants from all over the country and providing a representative sample, are needed.

## Figures and Tables

**Table 1:** Average adherence scores per participants based on demographic characteristics.

Patients’ Characteristics	Number of Participants (%)	Average Adherence SEAMS Score (SD)
Patients’ gender		
• male	115 (49.6)	49.14 (SD 7.59)
• female	117 (50.4)	48.94 (SD 8.44)
Patients’ education		
• primary school	18 (7.8)	47.86 (SD 10.16)
• secondary school	129 (55.6)	48.86 (SD 9.91)
• university (master’s and bachelor’s degree)	85 (36.6)	48.26 (SD 9.92)
Co-morbidity (number of diagnoses in addition to hypertension)		
• one disease	49 (21.1)	48.78 (SD 9.97)
• two diseases	66 (28.4)	48.17 (SD 9.92)
• three diseases	63 (27.1)	48.16 (SD 9.96)
• four diseases	31(13.4)	48.02 (SD 10.05)
• five diseases	18 (7.7)	47.77 (SD 10.13)
• six diseases	4 (1.7)	46.92 (SD 10.91)
• seven diseases	1 (0.5)	52.00 *
Number of prescribed medicines		
• non-pharmacological	4 (1.7)	52.25 (SD 10.75)
• one medicinal product	36 (15.5)	50.84 (SD 10.03)
• two medicines	40 (17.2)	48.63 (SD 9.85)
• three medicines	42 (18.1)	49.64 (SD 9.89)
• four medicines	38 (16.4)	47.74 (SD 9.93)
• five medicines	25 (10.8)	46.97 (SD 10.04)
• six medicines	23 (9.9)	48.00 (SD 10.07)
• seven medicines	15 (6.5)	48.47 (SD 10.44)
• eight medicines	6 (2.6)	51.50 (SD 10.44)
• nine medicines	2 (0.9)	48.50 *
• eleven medicines	1 (0.5)	49.00 *

* indicates that due to the small number of participants, SD is not calculated.

**Table 2:** Covariance results, mean value, and SD per question.

SEAMS Questions	Covariance Result	Mean	SD
When you take several different medicines each day?	−0.08102	2.78	0.504
When you have a busy day planned?	0.000907	2.73	0.527
When you are away from home?	−0.05626	2.71	0.535
When no one reminds you to take the medicine?	−0.05852	2.68	0.594
When you take medicines more than once a day?	−0.00881	2.71	0.567
When the schedule to take the medicine is not convenient?	−0.0339	2.59	0.619
When your normal routine gets messed up?	−0.02219	2.64	0.582
When you get a refill of your old medicines and some of the pills look different than usual?	−0.12987	2.46	0.747
When you are not sure how to take the medicine?	−0.12442	2.34	0.754
When you are not sure what time of the day to take your medicine?	−0.06575	2.37	0.706
When a doctor changes your medicines?	0.039397	2.62	0.607
When they cause some side effects?	−0.14002	2.15	0.883
When you are feeling sick (like having a cold or the flu)?	−0.06582	2.52	0.693
When you are feeling fine?	−0.05355	2.68	0.601
Fill your prescriptions whatever they cost?	−0.04473	2.50	0.718
Take your medicines for the rest of your life?	−0.05682	2.76	0.495
How confident are you that you will be able to take all or most of your medicines as directed?	−0.17119	2.69	0.573
Make taking your medicines part of your routine?	0.073573	2.72	0.569
Always remember to take your medicines?	0.133026	2.46	0.747

**Table 3:** The correlation between adherence results and patients’ age, an average number of diagnoses, and prescribed medicines (SEAMS).

Patients’Age	Number of Responded Patients	An Average Number of Diagnoses per Patient	An Average Number of Prescribed Medicinal Products	% Patients Treated with FDC (of the Total Number of Patients in the Group)	An Average Adherence Score of Patients Treated with FDC	An Average Adherence Score of Patients Treated with Mono-Products	An Average Adherence Score
To20	1	NA	1	NA	NA	55.00	NA
20–29	9	1.78	1.44	11.11	48.00	55.43	51.72
30–39	16	1.59	1.82	23.53	47.00	53.50	50.25
40–49	35	2.06	2.41	45.71	51.06	48.00	49.53
50–59	36	2.31	3.34	63.89	52.04	52.70	52.37
60–69	61	2.72	4.08	68.85	47.02	45.11	46.07
70–79	53	3.13	4.82	49.06	48.00	47.23	47.612
80–89	18	3.83	4.94	61.11	46.80	52.57	49.69
Above 90	3	4.33	5.33	NA	57.00	46.50	51.75

NA—an average value (or %) cannot be calculated.

## Data Availability

The data that support the findings of this study are available upon reasonable request from the corresponding author (Z.M.).

## Appendix A. SEAM Questionnaire

SEAMS Questions	Not at All	Sometimes	Completely
How confident are you:	1	2	3
When you take several different medicines each day?			
When you have a busy day planned?			
When you are away from home			
When no one reminds you to take the medicine?			
When you take medicines more than once a day?			
When the schedule to take the medicine is not convenient?			
When your normal routine gets messed up?			
When you get a refill of your old medicines and some of the pills look different than usual?			
When you are not sure how to take the medicine?			
When you are not sure what time of the day to take your medicine?			
When a doctor changes your medicines?			
When they cause some side effects?			
When you are feeling sick (like having a cold or the flu)?			
When you are feeling fine?			
Fill your prescriptions whatever they cost?			
Take your medicines for the rest of your life?			
How confident are you that you will be able to take all or most of your medicines as directed?			
Make taking your medicines part of your routine?			
Always remember to take your medicines?			
